# 5-(4-Bromo­benzyl­idene)-5*H*-dibenzo[*a*,*d*][7]annulene

**DOI:** 10.1107/S1600536808003711

**Published:** 2008-02-15

**Authors:** Ren-Hua Zheng

**Affiliations:** a School of Pharmaceutical and Chemical Engineering, Taizhou University, Linhai 317000, People’s Republic of China

## Abstract

The tricyclic system of the title compound, C_22_H_15_Br, has a concave shape, with a dihedral angle between the benzene ring planes of 48.68 (1)°.

## Related literature

For related literature, see: Allen *et al.* (1987 ▶); Bergmann & Solomonovici (1970 ▶); Larson (1970 ▶).
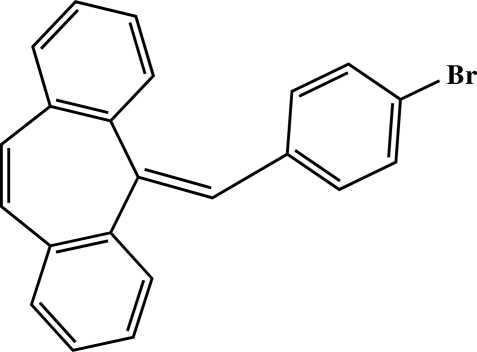


## Experimental

### 

#### Crystal data

C_22_H_15_Br
*M* *_r_* = 359.26
Monoclinic, 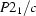

*a* = 8.4857 (5) Å
*b* = 19.0479 (8) Å
*c* = 10.6808 (5) Å
β = 104.6802 (16)°
*V* = 1670.03 (14) Å^3^
*Z* = 4
Mo *K*α radiation
μ = 2.46 mm^−1^
*T* = 296 (1) K
0.57 × 0.46 × 0.29 mm

#### Data collection

Rigaku R-AXIS RAPID diffractometer
Absorption correction: multi-scan (*ABSCOR*; Higashi, 1995 ▶) *T*_min_ = 0.227, *T*_max_ = 0.489
8896 measured reflections
3792 independent reflections
2462 reflections with *F* ^2^ > 2σ(*F* ^2^)
*R*_int_ = 0.051

#### Refinement

*R*[*F* ^2^ > 2σ(*F* ^2^)] = 0.053
*wR*(*F* ^2^) = 0.140
*S* = 1.00
3792 reflections
209 parameters
H-atom parameters constrained
Δρ_max_ = 0.83 e Å^−3^
Δρ_min_ = −0.93 e Å^−3^

### 

Data collection: *PROCESS-AUTO* (Rigaku, 1998 ▶); cell refinement: *PROCESS-AUTO*; data reduction: *CrystalStructure* (Rigaku/MSC, 2004 ▶); program(s) used to solve structure: *SIR97* (Altomare *et al.*, 1999 ▶); program(s) used to refine structure: *CRYSTALS* (Betteridge *et al.*, 2003 ▶); molecular graphics: *ORTEP-3 for Windows* (Farrugia, 1997 ▶); software used to prepare material for publication: *CrystalStructure*.

## Supplementary Material

Crystal structure: contains datablocks globbal, I. DOI: 10.1107/S1600536808003711/gk2131sup1.cif

Structure factors: contains datablocks I. DOI: 10.1107/S1600536808003711/gk2131Isup2.hkl

Additional supplementary materials:  crystallographic information; 3D view; checkCIF report

## Figures and Tables

**Fig. 1.**
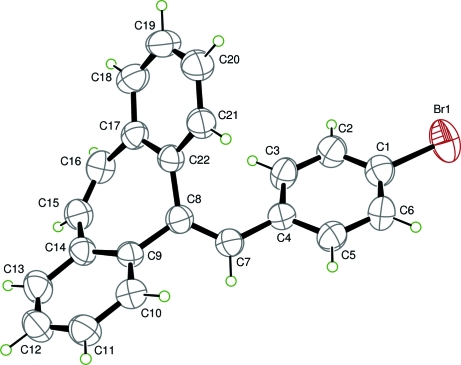
Molecular structure of of the title compound with displacement ellipsoids shown at the 50% probability level.

**Fig. 2.**
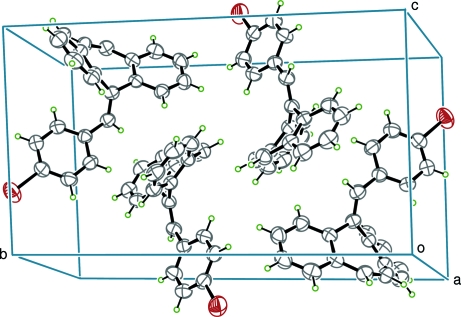
Crystal packing diagram of the title compound.

## supplementary crystallographic information

### Comment

The title compound was synthesized through Wittig–Horner reaction (Bergmann & Solomonovici, 1970).

The molecular structure is shown in Fig. 1. The bond lengths and angles are generally within normal ranges (Allen *et al.*,1987). Packing diagram is given in Fig. 2. The seven-membered ring is in a boat conformation. The dihedral angle between the benzene A (C1–C6) and the plane defined by the atoms C9/C14/C17 /C22 is 29.5 (1)°. Benzene C (C9–C14) and benzene D (C17–C22) form with the plane defined by C9/C14/C17/C22 dihedral angles of 24.1 (1)° and 26.4 (1)°, respectively, while the dihedral angle between them is 48.68 (1)°. The crystal packing (Fig. 2) is mainly stabilized by van der Waals forces.

### Experimental

The title compound was synthesized by treating solution of (4-bromo-benzyl)-phosphonic acid diethyl ester (1.53 g, 5 mmol) and dibenzo[a,d]cyclohepten-5-one (1.03 g, 5 mmol) in 100 ml anhydrous THF under nitrogen with solid potassium *tert*-butoxide (1.68 g, 15 mmol) which was added in one portion. The mixture was refluxed with stirring for 10 h. Solvent was removed by rotary evaporation. The residue was purified by column chromatography (silica gel) using n-hexane as eluent. Colorless crystals were obtained by slow evaporation of a dichloromethane solution.

### Refinement

The H atoms were positioned geometrically and allowed to ride on their parent atoms, with C—H = 0.93 Å and *U*_iso_ = 1.2Ueq(C).

### Crystal data

**Table:**

C_22_H_15_Br	*F*_000_ = 728.00
*M**_r_* = 359.26	*D*_x_ = 1.429 Mg m^−^^3^
Monoclinic, *P*2_1_/*c*	Mo *K*α radiation λ = 0.71075 Å
Hall symbol: -P 2ybc	Cell parameters from 8966 reflections
*a* = 8.4857 (5) Å	θ = 3.2–27.4º
*b* = 19.0479 (8) Å	µ = 2.47 mm^−^^1^
*c* = 10.6808 (5) Å	*T* = 296 (1) K
β = 104.6802 (16)º	Chunk, colourless
*V* = 1670.03 (14) Å^3^	0.57 × 0.46 × 0.29 mm
*Z* = 4	

### Data collection

**Table:**

Rigaku R-AXIS RAPID diffractometer	2462 reflections with *F*^2^ > 2σ(*F*^2^)
Detector resolution: 10.00 pixels mm^-1^	*R*_int_ = 0.051
ω scans	θ_max_ = 27.4º
Absorption correction: multi-scan(ABSCOR; Higashi, 1995)	*h* = 0→10
*T*_min_ = 0.227, *T*_max_ = 0.489	*k* = 0→24
8896 measured reflections	*l* = −13→13
3792 independent reflections	

### Refinement

**Table:**

Refinement on *F*^2^	*w* = 1/[0.001*F*_o_^2^ + 6.2σ(*F*_o_^2^)]/(4*F*_o_^2^)
*R*[*F*^2^ > 2σ(*F*^2^)] = 0.053	(Δ/σ)_max_ < 0.001
*wR*(*F*^2^) = 0.140	Δρ_max_ = 0.83 e Å^−^^3^
*S* = 1.01	Δρ_min_ = −0.93 e Å^−^^3^
3792 reflections	Extinction correction: Larson (1970)
209 parameters	Extinction coefficient: 351 (32)
H-atom parameters constrained	

### Special details

**Table:**

Refinement. Refinement using all reflections. The weighted *R*-factor (*wR*) and goodness of fit (*S*) are based on *F*^2^. *R*-factor (gt) are based on *F*. The threshold expression of *F*^2^ > 2.0 σ(*F*^2^) is used only for calculating *R*-factor (gt).

### Fractional atomic coordinates and isotropic or equivalent isotropic displacement parameters (Å^2^)

**Table:**

	*x*	*y*	*z*	*U*_iso_*/*U*_eq_	
Br1	0.09761 (6)	0.48750 (2)	−0.21051 (4)	0.08526 (18)	
C1	0.2486 (4)	0.54224 (18)	−0.0874 (3)	0.0574 (10)	
C2	0.2994 (4)	0.51963 (18)	0.0385 (3)	0.0618 (11)	
C3	0.4098 (4)	0.55959 (17)	0.1270 (3)	0.0586 (11)	
C4	0.4709 (4)	0.62248 (17)	0.0924 (2)	0.0482 (9)	
C5	0.4180 (4)	0.64341 (19)	−0.0356 (3)	0.0588 (11)	
C6	0.3069 (4)	0.60356 (19)	−0.1258 (3)	0.0616 (11)	
C7	0.5914 (4)	0.66577 (17)	0.1840 (3)	0.0516 (10)	
C8	0.6033 (4)	0.67805 (17)	0.3102 (2)	0.0481 (9)	
C9	0.7365 (3)	0.72298 (16)	0.3866 (2)	0.0450 (9)	
C10	0.7624 (4)	0.78939 (18)	0.3398 (3)	0.0561 (10)	
C11	0.8893 (4)	0.83153 (19)	0.4062 (3)	0.0612 (11)	
C12	0.9902 (4)	0.8091 (2)	0.5201 (3)	0.0654 (12)	
C13	0.9669 (4)	0.7436 (2)	0.5676 (3)	0.0618 (11)	
C14	0.8397 (4)	0.69947 (17)	0.5039 (3)	0.0502 (9)	
C15	0.8235 (4)	0.63084 (18)	0.5605 (3)	0.0560 (10)	
C16	0.6901 (4)	0.59282 (18)	0.5526 (3)	0.0573 (10)	
C17	0.5221 (4)	0.61096 (17)	0.4876 (2)	0.0493 (9)	
C18	0.3957 (5)	0.58558 (19)	0.5374 (3)	0.0643 (12)	
C19	0.2344 (5)	0.6034 (2)	0.4844 (4)	0.0720 (14)	
C20	0.1960 (5)	0.6485 (2)	0.3806 (3)	0.0673 (13)	
C21	0.3170 (4)	0.67289 (18)	0.3276 (3)	0.0550 (10)	
C22	0.4800 (4)	0.65368 (14)	0.3770 (2)	0.0459 (9)	
H2	0.2599	0.4778	0.0637	0.074*	
H3	0.4444	0.5441	0.2121	0.070*	
H5	0.4576	0.6850	−0.0618	0.071*	
H6	0.2725	0.6183	−0.2114	0.074*	
H7	0.6696	0.6875	0.1501	0.062*	
H10	0.6935	0.8055	0.2630	0.067*	
H11	0.9060	0.8753	0.3731	0.073*	
H12	1.0740	0.8378	0.5654	0.078*	
H13	1.0377	0.7283	0.6441	0.074*	
H15	0.9200	0.6110	0.6087	0.067*	
H16	0.7057	0.5493	0.5935	0.069*	
H18	0.4209	0.5557	0.6086	0.077*	
H19	0.1528	0.5850	0.5187	0.086*	
H20	0.0886	0.6624	0.3465	0.081*	
H21	0.2898	0.7030	0.2568	0.066*	

### Atomic displacement parameters (Å^2^)

**Table:**

	*U*^11^	*U*^22^	*U*^33^	*U*^12^	*U*^13^	*U*^23^
Br1	0.0863 (4)	0.0690 (3)	0.0828 (3)	−0.0041 (2)	−0.0113 (2)	−0.0165 (2)
C1	0.061 (2)	0.0547 (19)	0.057 (2)	0.0027 (17)	0.0150 (18)	−0.0078 (16)
C2	0.075 (2)	0.0516 (19)	0.059 (2)	−0.0067 (18)	0.0163 (19)	−0.0031 (16)
C3	0.072 (2)	0.058 (2)	0.0442 (19)	−0.0005 (18)	0.0113 (18)	0.0038 (15)
C4	0.047 (2)	0.0511 (17)	0.0476 (18)	−0.0003 (15)	0.0138 (15)	−0.0006 (14)
C5	0.065 (2)	0.060 (2)	0.050 (2)	−0.0063 (18)	0.0130 (18)	0.0094 (15)
C6	0.068 (2)	0.067 (2)	0.046 (2)	0.0006 (19)	0.0055 (18)	−0.0010 (16)
C7	0.047 (2)	0.0579 (19)	0.0488 (19)	−0.0013 (15)	0.0106 (16)	0.0042 (15)
C8	0.045 (2)	0.0513 (18)	0.0462 (19)	0.0033 (14)	0.0092 (15)	0.0039 (14)
C9	0.0390 (18)	0.0527 (17)	0.0451 (17)	−0.0027 (14)	0.0137 (14)	−0.0061 (14)
C10	0.054 (2)	0.062 (2)	0.053 (2)	−0.0040 (17)	0.0153 (17)	0.0009 (16)
C11	0.059 (2)	0.059 (2)	0.067 (2)	−0.0109 (18)	0.0178 (19)	−0.0059 (17)
C12	0.054 (2)	0.072 (2)	0.069 (2)	−0.0145 (18)	0.012 (2)	−0.0151 (19)
C13	0.046 (2)	0.076 (2)	0.057 (2)	0.0030 (18)	0.0017 (17)	−0.0032 (18)
C14	0.0395 (19)	0.0592 (19)	0.0508 (19)	0.0018 (15)	0.0095 (15)	−0.0089 (15)
C15	0.052 (2)	0.058 (2)	0.054 (2)	0.0050 (17)	0.0062 (17)	0.0034 (16)
C16	0.068 (2)	0.0480 (18)	0.053 (2)	0.0076 (17)	0.0106 (18)	0.0045 (14)
C17	0.056 (2)	0.0462 (17)	0.0474 (19)	−0.0015 (15)	0.0160 (17)	−0.0048 (13)
C18	0.074 (2)	0.065 (2)	0.060 (2)	−0.005 (2)	0.028 (2)	0.0046 (17)
C19	0.065 (2)	0.086 (2)	0.075 (2)	−0.017 (2)	0.036 (2)	−0.012 (2)
C20	0.051 (2)	0.084 (2)	0.069 (2)	0.002 (2)	0.021 (2)	−0.016 (2)
C21	0.050 (2)	0.062 (2)	0.053 (2)	0.0002 (17)	0.0117 (17)	−0.0066 (16)
C22	0.046 (2)	0.0461 (17)	0.0466 (17)	−0.0028 (14)	0.0123 (15)	−0.0064 (13)

### Geometric parameters (Å, °)

**Table:**

Br1—C1	1.899 (3)	C17—C22	1.404 (4)
C1—C2	1.372 (4)	C18—C19	1.384 (5)
C1—C6	1.371 (5)	C19—C20	1.375 (5)
C2—C3	1.379 (4)	C20—C21	1.374 (6)
C3—C4	1.392 (4)	C21—C22	1.399 (4)
C4—C5	1.386 (4)	C2—H2	0.930
C4—C7	1.475 (4)	C3—H3	0.930
C5—C6	1.390 (4)	C5—H5	0.930
C7—C8	1.346 (4)	C6—H6	0.930
C8—C9	1.486 (4)	C7—H7	0.930
C8—C22	1.482 (5)	C10—H10	0.930
C9—C10	1.398 (4)	C11—H11	0.930
C9—C14	1.408 (4)	C12—H12	0.930
C10—C11	1.386 (4)	C13—H13	0.930
C11—C12	1.367 (4)	C15—H15	0.930
C12—C13	1.378 (5)	C16—H16	0.930
C13—C14	1.401 (4)	C18—H18	0.930
C14—C15	1.461 (4)	C19—H19	0.930
C15—C16	1.328 (5)	C20—H20	0.930
C16—C17	1.461 (4)	C21—H21	0.930
C17—C18	1.398 (6)		
			
Br1—C1—C2	119.7 (2)	C8—C22—C17	121.8 (2)
Br1—C1—C6	119.4 (2)	C8—C22—C21	119.2 (2)
C2—C1—C6	120.9 (3)	C17—C22—C21	119.1 (3)
C1—C2—C3	119.2 (3)	C1—C2—H2	120.4
C2—C3—C4	121.8 (3)	C3—C2—H2	120.4
C3—C4—C5	117.4 (2)	C2—C3—H3	119.1
C3—C4—C7	123.2 (2)	C4—C3—H3	119.1
C5—C4—C7	119.4 (3)	C4—C5—H5	119.3
C4—C5—C6	121.3 (3)	C6—C5—H5	119.3
C1—C6—C5	119.3 (3)	C1—C6—H6	120.3
C4—C7—C8	128.7 (3)	C5—C6—H6	120.3
C7—C8—C9	120.2 (3)	C4—C7—H7	115.7
C7—C8—C22	123.0 (2)	C8—C7—H7	115.7
C9—C8—C22	116.6 (2)	C9—C10—H10	119.6
C8—C9—C10	119.4 (2)	C11—C10—H10	119.6
C8—C9—C14	121.4 (2)	C10—C11—H11	119.9
C10—C9—C14	119.2 (2)	C12—C11—H11	119.9
C9—C10—C11	120.9 (2)	C11—C12—H12	120.2
C10—C11—C12	120.3 (3)	C13—C12—H12	120.2
C11—C12—C13	119.7 (3)	C12—C13—H13	119.0
C12—C13—C14	121.9 (3)	C14—C13—H13	119.0
C9—C14—C13	118.1 (3)	C14—C15—H15	115.5
C9—C14—C15	123.4 (2)	C16—C15—H15	115.5
C13—C14—C15	118.5 (2)	C15—C16—H16	116.0
C14—C15—C16	129.0 (3)	C17—C16—H16	116.0
C15—C16—C17	127.9 (3)	C17—C18—H18	118.9
C16—C17—C18	119.2 (3)	C19—C18—H18	118.9
C16—C17—C22	123.1 (3)	C18—C19—H19	120.3
C18—C17—C22	117.7 (3)	C20—C19—H19	120.3
C17—C18—C19	122.2 (3)	C19—C20—H20	120.2
C18—C19—C20	119.4 (4)	C21—C20—H20	120.2
C19—C20—C21	119.7 (3)	C20—C21—H21	119.1
C20—C21—C22	121.8 (3)	C22—C21—H21	119.1
			
Br1—C1—C2—C3	179.7 (3)	C10—C9—C14—C13	−1.3 (5)
Br1—C1—C6—C5	−179.7 (3)	C10—C9—C14—C15	−179.6 (3)
C2—C1—C6—C5	−0.5 (6)	C14—C9—C10—C11	1.0 (5)
C6—C1—C2—C3	0.4 (6)	C9—C10—C11—C12	−1.0 (6)
C1—C2—C3—C4	0.2 (5)	C10—C11—C12—C13	1.2 (6)
C2—C3—C4—C5	−0.7 (5)	C11—C12—C13—C14	−1.5 (6)
C2—C3—C4—C7	−178.8 (3)	C12—C13—C14—C9	1.5 (5)
C3—C4—C5—C6	0.7 (5)	C12—C13—C14—C15	180.0 (2)
C3—C4—C7—C8	−37.4 (5)	C9—C14—C15—C16	−30.8 (6)
C5—C4—C7—C8	144.6 (3)	C13—C14—C15—C16	150.9 (4)
C7—C4—C5—C6	178.8 (3)	C14—C15—C16—C17	−2.0 (6)
C4—C5—C6—C1	−0.1 (4)	C15—C16—C17—C18	−148.6 (3)
C4—C7—C8—C9	179.2 (3)	C15—C16—C17—C22	30.7 (5)
C4—C7—C8—C22	−6.3 (5)	C16—C17—C18—C19	176.9 (3)
C7—C8—C9—C10	51.4 (4)	C16—C17—C22—C8	6.1 (4)
C7—C8—C9—C14	−127.2 (3)	C16—C17—C22—C21	−174.9 (3)
C7—C8—C22—C17	123.5 (3)	C18—C17—C22—C8	−174.6 (3)
C7—C8—C22—C21	−55.6 (4)	C18—C17—C22—C21	4.5 (4)
C9—C8—C22—C17	−61.8 (4)	C22—C17—C18—C19	−2.5 (5)
C9—C8—C22—C21	119.2 (3)	C17—C18—C19—C20	−1.2 (6)
C22—C8—C9—C10	−123.5 (3)	C18—C19—C20—C21	2.8 (6)
C22—C8—C9—C14	57.9 (4)	C19—C20—C21—C22	−0.7 (5)
C8—C9—C10—C11	−177.6 (3)	C20—C21—C22—C8	176.0 (3)
C8—C9—C14—C13	177.3 (3)	C20—C21—C22—C17	−3.1 (4)
C8—C9—C14—C15	−1.0 (5)		
